# Stayability in the Era of Early‐Challenged Females: Genetic Parameters and Correlations With Economically Relevant Traits

**DOI:** 10.1111/jbg.70026

**Published:** 2025-11-24

**Authors:** Letícia Silva Pereira, Larissa Bordin Temp, Gabriel Gubiani, Miller Teodoro, Cláudio Ulhoa Magnabosco, Fernando Baldi

**Affiliations:** ^1^ National Association of Breeders and Researchers (ANCP) Ribeirão Preto SP Brazil; ^2^ Department of Animal Science, College of Agricultural and Veterinary Sciences São Paulo State University (UNESP) Jaboticabal SP Brazil; ^3^ Embrapa Cerrados Brasília DF Brazil; ^4^ Department of Animal Science, Faculty of Animal Science and Food Engineering University of São Paulo São Paulo Brazil

**Keywords:** longevity, Nellore, selection criteria, sexual precocity

## Abstract

This study aimed to estimate the variance components, heritabilities and genetic correlations between four new different categories of stayability (STAY48‐2, STAY48‐3, STAY54‐2, STAY54‐3) with weight at 240 days of age (W240), weight at 450 days of age (W450), scrotal circumference at 365 days of age (SC365), age at puberty in males (APM), traditional stayability (STAY72), probability of precocious calving at 30 months of age (PPC30), ribeye area (REA), rump fat thickness (RFT), residual feed intake (RFI), dry‐matter intake (DMI), residual live weight gain (RG) and frame score (FRAME). Records from 80,958 females born between 2000 and 2019, exposed to mating starting at 10 months of age, raised on pasture from 508 farms in the central‐west, southeast, northeast and northern regions of Brazil that participate in the National Association of Breeders and Researchers (ANCP), were analysed. The (co)variance components were estimated by Bayesian inference in a two‐trait animal model. The *posterior* means of heritability estimates for STAY48‐2, STAY48‐3, STAY54‐2 and STAY54‐3 were moderate to low, 0.20, 0.20, 0.22 and 0.22 respectively. The traits related to different categories of stayability showed low genetic correlations with male reproductive traits (−0.22 to 0.19), feed efficiency (−0.03 to 0.13), carcass (0.11 to 0.18) and body composition (−0.09 to −0.6), moderate with growth (0.04 to 0.29) and high with sexual precocity (0.88 to 0.93) and fertility (0.65 to 0.73). The heritability estimates of the different categories of stayability indicate genetic improvement for longevity in early challenged females. The genetic correlations with sexual precocity showed that its use as selection criteria is recommended for production systems of younger females challenged in the reproductive season rather than traditional stayability to increase the probability of stayability in the herd.

## 1 Introduction

The profitability of beef cattle herds is directly related to the productivity of the dams, measured by the number of calves weaned per cow, and their longevity in the herd.

For commercial cow–calf operators, producing a healthy, quality calf each season is a key objective, and no component is as crucial as the reproductive efficiency of the heifers (Martinez et al. 2005). Raising heifers requires considerable time and resources (Martinez et al. 2005). To achieve economic success, the producer must maintain the replacement heifers in the production system for a period necessary to produce enough calves to offset the maintenance costs of the replacements themselves (Doyle et al. 2000). In this regard, reproductive traits have a greater impact on the herd compared to growth traits (Silva et al. 2024).

Among these, stayability (STAY) stands out as one of the most economically important traits for herd longevity (Shabalina et al. 2020). It is defined as the probability of a cow remaining productive in the herd until a specific age and producing a defined number of calves, since the dam has the opportunity or chance to reach that age (Hudson and Van Vleck 1981). The measurement of STAY is more complex than other traits due to its categorical trait, low heritability and strong environmental influence (Schmidt et al. 2018; Silva Neto et al. 2020; Negreiros et al. 2022) and its evaluation being limited to females during their productive cycle in Nellore cattle. STAY has been defined as the production of three calves up to 72 months of age in Zebubreed selection programs (Lôbo et al. 2024).

The traditional measurement at 72 months of age limits the number of females evaluated, as younger animals do not have data available until they reach this age (Ramos et al. 2020). Another important aspect is that this long age of 76 months allows females to have up to two reproductive failures, which can lead to the selection of dams with low fertility at the beginning of their reproductive life (Ramos et al. 2020). In seedstock herds, using genomics and earlier selection, the turnover of dams is higher, and cows do not always reach 76 months of age due to a reduction in culling age, thereby decreasing the average age of the herd.

The increasing frequency of heifers expressing early sexual precocity in herds has emerged as a promising strategy, resulting in more productive dams, a greater number of calves throughout their lifetime and higher economic returns for producers (Terakado et al. 2015). In Brazil, research on sexual precocity in Zebu females has promoted considerable genetic progress for this trait in heifers exposed to the reproductive season between 11 and 15 months of age (Bonamy et al. 2018; Fernandes Júnior et al. 2022; Terakado et al. 2015), especially in the Nellore breed, over the last 14 years, accompanied by a reflection on the increase in the number of herds that adopt this practice. In this sense, traditional STAY for these precocious females may not be the most appropriate criterion to reflect their fertility and the longevity of their reproductive life.

To speed up genetic gain and address the need for a STAY trait that reflects these earlier‐maturing females, measuring the trait at younger ages presents a viable alternative (Ramos et al. 2020). In this context, new STAY criteria are proposed for these heifers that begin their reproductive life earlier, such as evaluating this trait at 48 or 54 months of age, defined as the ability of females to remain in the herd until these ages and produce at least two or three calves. These newly proposed criteria are probably more associated with sexual precocity, the ability to remain and reconception, that is, the return to reproductive activity earlier after the first calving. This is particularly crucial in herds and selection programs due to the reduction in calving intervals, increasing reproductive efficiency and productivity throughout the female's life, as well as promoting greater genetic progress for this trait, directly impacting the economic sustainability of the production system.

The sustainability of beef production systems, both biological and economic, depends on improving key traits such as animal growth, reproductive performance, carcass yield and meat quality (Mamede et al. 2023). In the beef industry, growth and carcass traits play a crucial role in profitability, making their inclusion in Nellore breeding programs essential for enhancing production efficiency and product quality. Furthermore, reproduction and longevity traits, such as STAY, are equally important for ensuring long‐term productivity. Identifying the genetic associations among growth, carcass, reproduction and production traits is vital for incorporating STAY into selection strategies, particularly for early sexual precocious heifers. According to Ochsner et al. (2017), estimating genetic parameters is fundamental for developing reliable selection indices, enabling the simultaneous selection of multiple traits and using STAY to indicate female longevity in early sexual precocious heifers.

The aims of the study were: (1) To estimate the (co)variance components and genetic parameters for the new STAY criteria for two or three calving up to 48 and 54 months (STAY48‐2, STAY48‐3, STAY54‐2 and STAY54‐3); (2) to compare traditional stayability measured at 72 months, with the new stayability criteria and (3) to assess genetic correlations between stayability for early‐challenged females with growth, fertility, sexual precocity, carcass, feed efficiency and body composition traits in females of the Nellore breed.

## 2 Materials and Methods

### 2.1 Database Description

This study was conducted using a dataset on growth, fertility, sexual precocity, carcass traits, feed efficiency and body composition from herds enrolled in the Nellore Brazil Genetic Improvement Program, coordinated by the National Association of Breeders and Researchers (ANCP). The animals belonged to farms in Brazil's central‐west, southeast, northeast and northern regions.

### 2.2 Evaluated Taits

The traits related to growth in this study were adjusted weight at 240 days of age (W240 kg) and adjusted weight at 450 days of age (W450 kg). Standardised weights were calculated using linear regression, considering the average daily gain between 165 and 255 and 405 and 495 days of age for W240 and W450 respectively (Negreiros et al. 2022). The carcass traits considered were ribeye area (REA, cm^2^) and rump fat thickness (RFT, mm). Carcass phenotypes were collected through ultrasound images obtained from the *Longissimus dorsi* muscle between the 12th and 13th ribs (REA) and in the rump region, between the ilium and ischium at the intersection of the *Gluteus medius* and *Biceps femoris* (RFT) muscles, using the ALOKA 500 V equipment with a 3.5 MHz linear probe. The age range of the animals at the time of data collection was from 304 to 787 days. Stayability (STAY, %) was considered in this study. To calculate traditional stayability, all females in the database were used, regardless of whether they had been challenged or not. To calculate the new stayability approaches in early sexually precocious heifers, only challenged females were used. For STAY, females born between 2000 and 2019 were classified according to the number of calvings at different ages, as described below:

#### 2.2.1 STAY48‐2

Challenged females that produced two calves by 48 months of age received a score of 2 (success), while those that did not meet the previous criterion received a score of 1 (failure).

#### 2.2.2 STAY48‐3

Challenged females that produced three calves by 48 months of age received a score of 2 (success), while those that did not meet the previous criterion received a score of 1 (failure).

#### 2.2.3 STAY54‐2

Challenged females that produced two calves by 54 months of age received a score of 2 (success), while those that did not meet the previous criterion received a score of 1 (failure).

#### 2.2.4 STAY54‐3

Challenged females that produced three calves by 54 months of age received a score of 2 (success), while those that did not meet the previous criterion received a score of 1 (failure).

#### 2.2.5 Traditional STAY72

Challenged females that produced 3 calves by 72 months of age were given a score of 2 (success), while those that did not meet the above criterion were given a score of 1 (failure).

The traits indicating sexual precocity considered in this study were the probability of early calving (PPC30, %) and the age at puberty for males (APM). All heifers that underwent the sexual precocity program were exposed to reproduction during the weaning year as part of the PPC30 determination process. Those females that confirmed pregnancy and gave birth to a live calf by 30 months of age received a score of 2 (success), while the others that failed received a score of 1 (failure). The bulls underwent testicular ultrasonography and andrological clinical examination to determine the males' puberty age. According to the assessment, the animals were categorised as super‐early (pubertal at ≤ 14 months of age), early (puberty between 14 and 17 months of age) or traditional (puberty > 17 months of age) (Silva et al. 2024). Scrotal circumference adjusted at 365 days of age (SC365) was measured in centimetres with a measuring tape, from 9 to 18 months of age in intervals of 3 months.

The traits of feed efficiency, residual feed intake (RFI kg of dry matter/day), dry matter intake (DMI kg/day) and residual body weight gain (RG) were obtained through Intergado and GrowSafe electronic systems. Feed efficiency tests followed the guidelines established by Mendes et al. (2020) for assessing individual feed intake in beef cattle using both electronic systems. Animals were kept in collective or individual pens and subjected to a 21 day adaptation period followed by a valid 70 day testing phase. Throughout this period, each animal's average weight was recorded via manual weighing every 14 days or through automated weighing platforms (Intergado).

To obtain RFI, the average daily gain (ADG kg/day) and metabolic live weight (MW^*75*^) were calculated. Daily dry matter intake was derived from the means of all valid individual daily intake values electronically recorded by the Intergado and GrowSafe systems during the test period. ADG was estimated using a linear regression coefficient of animal's weight on the days in the test for the animals (Koch et al. 1963): $\mathrm{ADG}=\alpha +\beta {\mathrm{DET}}_{i}+\varepsilon_{i}$ (1), where $y_{i}$ is the animal's weight, α is the intercept of the regression equation that represents the initial weight, β is the linear regression coefficient that represents ADG, ${\mathrm{DET}}_{i}$ are the days in the performance test of the *n* ^th^ observation and $\varepsilon_{i}$ is the residual associated with each observation.

Using the estimated coefficients from (1) (MW^0.75^), was calculated using the formula (Koch et al. 1963): ${\mathrm{MW}}^{0.75}={\left[\hat{\alpha }+\hat{\beta }\left(\frac{{\mathrm{DET}}_{i}}{2}\right)\right]}^{0.75}$ (2), where $\hat{\alpha }$ is live weight at the beginning of the feed efficiency test, $\hat{\beta }$ is the ADG.

RFI was calculated as the difference between predicted and observed dry matter intake, using a regression equation based on MW^0.75^ and ADG, following the methodology proposed by Koch et al. (1963): $\mathrm{RFI}=\beta_{01}+\beta_{11}{\mathrm{ADG}}_{i}+\beta_{21}{{\mathrm{MW}}^{0.75}}_{i}+\varepsilon_{i1}\left(\mathrm{RFI}\right)$ (3), where **y** is the individual dry matter intake of the animal, $\beta_{01}$ is the intercept, $\beta_{11}$ and $\beta_{21}$ are the linear regression coefficients for ADG and ${\mathrm{MW}}^{0.75}$ and $\varepsilon_{i1}$ is the residual, which is equivalent to the RFI.

RG was calculated as the difference between the observed ADG and the estimated based on MW^0.75^ and DMI (Koch et al. 1963; Berry and Crowley 2012): $\mathrm{RG}=\beta_{02}+\beta_{12}{\mathrm{DMI}}_{i}+\beta_{22}{{\mathrm{MW}}^{0.75}}_{i}+\varepsilon_{i2}\left(\mathrm{RG}\right)$ (4), where $\beta_{02}$ is the intercept, $\beta_{12}$ and $\beta_{22}$ are the linear regression coefficients for DMI and ${\mathrm{MW}}^{0.75}$ and $\varepsilon_{i2}$ is the residual, which is the RG in this case. The calculation for the frame score was performed based on the equation developed by Guimarães et al. (2020), using the method of multiple linear regression prediction, applying different equations for males (1) and females (2), taking into account the relationship between height (HH), sex and age of the animal (AGE):


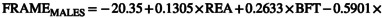
 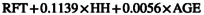 (5)


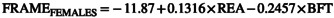
 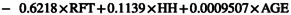 (6)

### 2.3 Statistical Analyses and Variance Components

The numerator relationship matrix contained 563,762 animals, including 19,358 sires and 291,365 dams. The animals that constituted the database had an average inbreeding coefficient of 1.04% and a proportion of 1.87% of inbred individuals in the total population. For STAY72 and different categories, the contemporary groups (CG) were created by concatenating farm, year and season of birth and animal management group, as shown in Table 1.

**TABLE 1:** Number of records (N), number of contemporary groups (CG) and percentage (%) of animals within each category for different categories of stayability trait in Nellore cattle.

Trait	N	CG	Mean (SD)	Min	Max	Success (2)	Failure (1)
STAY48‐2	80,957	1227	1.16 (0.37)	1.00	2.00	13,452 (16.62%)	65,505 (80.91%)
STAY48‐3	79,924	1203	1.15 (0.36)	1.00	2.00	12,163 (15.22%)	67,761 (84.78%)
STAY54‐2	75,101	1145	1.14 (0.35)	1.00	2.00	10,647 (14.18%)	64,454 (85.82%)
STAY54‐3	74,333	1126	1.13 (0.34)	1.00	2.00	10,304 (13.86%)	64,029 (86.14%)
STAY72–3	113,402	1901	1.36 (0.48)	1.00	2.00	41,300 (36.42%)	72,102 (63.58%)

*Note:* STAY48‐2: female's ability to remain in the herd up to 48 months of age, calving to at least 2 times; STAY48‐3: female's ability to remain in the herd up to 48 months of age, calving to at least 3 times, STAY54‐2: female's ability to remain in the herd up to 54 months of age, calving to at least 2 times; STAY54‐3: female's ability to remain in the herd up to 54 months of age, calving to at least 3 times and STAY72: female's ability to remain in the herd up to 72 months of age, calving to at least 3 times.

Abbreviation: SD: standard deviation.

For growth, feed efficiency, carcass fertility and sexual precocity traits of males CGs were formed by farm, year and season of birth, sex and animal management group. In addition, for feed efficiency traits, the efficiency test was added to its CG. For all traits, records within the mean ±3.0 standard deviations of the CG were maintained in the analysis. CGs with less than 20 and more than 80 animals were excluded for growth, carcass and scrotal circumference traits. For traits related to feed efficiency, CGs with less than 10 and more than 200 animals were removed. In the case of APM, CGs with less than 15 and more than 200 animals were excluded. For PPC30, only CGs with a minimum of 15 and a maximum of 150 animals were considered. For all stayability traits, CGs with less than 15 and more than 200 animals were excluded. Finally, CGs with less than 20 and more than 100 animals were removed for the FRAME trait. CGs without phenotypic variability for STAY and PPC30 were excluded. According to Van Vleck (1987), CGs that exhibit little or no variability, or that include only a small number of animals, may negatively influence the estimation of genetic parameters. Descriptive statistics for growth, carcass, sexual precocity, feed efficiency and body composition traits are shown in Table 2.

**TABLE 2:** Number of phenotypic records (N), contemporary groups (CG), mean, standard deviation (SD), minimum and maximum for growth, carcass, sexual precocity, feed efficiency and body composition traits on Nellore cattle.

Traits	N	CG	Mean (SD)	Minimum	Maximum
W240 (kg)	356,309	9207	204.47 (32.32)	24.00	348.00
W450 (kg)	250,296	9297	320.88 (61.99)	120.00	646.00
REA (cm^2^)	141,748	3785	61.09 (12.35)	18.74	128.01
RFT (mm)	141,533	3783	5.14 (2.67)	0.46	27.18
PPC30 (%)	92,817	1695	1.32 (0.46)	1.00	2.00
APM (months)	16,486	191	16.46 (3.63)	7.60	28.06
SC365 (cm)	110,947	4809	22.45 (2.70)	11.00	35.00
RFI (kg/day)	22.787	405	0.006 (0.82)	−7.10	6.94
DMI (kg/day)	22,787	405	8.44 (2.13)	2.52	20.65
RG (kg of ADG/day)	20,239	365	−0.005 (0.29)	−1.07	1.06
FRAME	39,439	969	5.78 (2.06)	−5.30	15.26

*Note:* W240: Adjusted weight at 240 days at age; W450: adjusted weigh at 450 days at age.

Abbreviations: ADG, average daily gain; APM, age at puberty in males; DMI, dry matter intake; FRAME, frame score; PPC30, probability of precocious calving before 30 months; REA, rib eye area; RFI, residual feed intake; RFT, rump fat thickness; RG, residual live weight gain; SC365, scrotal circumference adjusted at 365 days of age.

A threshold model was implemented via Bayesian inference, considering CG as a systematic effect and animal additive as random effects. The model for a latent variable (liability) is: l=Xβ+Zu+Mm+e (7), where: l is the vector of liability; **β** is the vector of fixed effects (CG); **u** is the vector of additive animal effects; **m** is the vector of maternal additive effect; **e** is the vector of residuals; **X, Z** and **M** are the incidence matrix that associates **β, u** and **m** to **l** respectively. Maternal genetic effect was considered only for W240. Given β, u and **m**, the elements of l are conditionally independent and as follows: $\left.l\right|\beta ,u,m,\sigma_{u}^{2},{\sigma_{m,}^{2}\sigma }_{e}^{2}\sim \mathrm{MVN}\left\{\mathrm{X\beta}+\mathrm{Zu}+\mathrm{Mm},I\sigma_{e}^{2}\right\}$. It was also assumed that: $\beta \sim \mathrm{MVN}\left\{0,I\sigma_{\beta }^{2}\right\}$; $u\sim \mathrm{MVN}\left\{0,A\sigma_{u}^{2}\right\}$ and $m\sim \mathrm{MVN}\left\{0,A\sigma_{m}^{2}\right\}$, where A is the numerator relationship matrix; and $e\sim \mathrm{MVN}\left\{0,I\sigma_{e}^{2}\right\}$, where I is an identity matrix of proper order; $\left.\sigma_{u}^{2}\right|\nu_{u}\sim \chi^{-2}\left(\nu_{u},S_{u}^{2}\right)$, $\left.\sigma_{m}^{2}\right|\nu_{m}\sim \chi^{-2}\left(\nu_{m},S_{m}^{2}\right)$ $\left.\sigma_{e}^{2}\right|\nu_{e}\sim \chi^{-2}\left(\nu_{e},S_{e}^{2}\right)$, being $\chi^{-2}$ an inverted chi‐square distribution with ν degrees of belief and S scale parameter matrix.

For all STAYs, the variance components were estimated via Gibbs sampling implemented in the GIBBSF90 + software (Misztal et al. 2014) using the default prior for random and systematic effects (i.e., using a Gaussian prior for random effects and a Uniform prior for systematic effects). In the Gibbs Sampling implementation, 300,000 iterations were employed, with an initial burn‐in of 100,000 iterations and a sampling interval of 100 iterations (thin). The POSTGIBBSF90 software (Misztal et al. 2014) was used for posterior inference.

Convergence was assessed through visual inspection of the generated Markov chains and by applying convergence diagnostics as follows: (1) Geweke's test (Geweke 1992), which compares the initial and final segments of the chain to detect convergence failures.

Rejection of the null hypothesis in this test indicates convergence, with probabilities below 0.05 providing evidence against chain convergence (Buzanskas et al. 2010); (2) Heidelberg and Welch test (Welch and Heidelberg 1983) was utilised, employing Cramer–von Mises statistics to evaluate the null hypothesis of stationarity in the generated samples and (3) Raftery and Lewis test (Raftery and Lewis 1992). All methods are implemented in the ‘boa’ R package (Smith 2005). Two‐trait analyses were performed to estimate genetic correlations between different criteria of stability and sexual precocity, fertility, growth, carcass, body composition and feed efficiency indicator traits using the BLUPF90 + program (Misztal et al. 2002).

## 3 Results and Discussion

The variance components and heritability estimates for the studied traits are presented in Table 3. The heritability estimates for the different STAY criteria were moderate, with values ranging from 0.20 to 0.22, while for traditional STAY, the heritability was lower at 0.16. These findings align with those reported by Silva et al. (2024) in Nellore cattle, where heritabilities ranged between 0.15 and 0.17 for the second and third calving, respectively, and 0.16 for traditional STAY (Bonamy et al. 2018).

**TABLE 3:** Posterior means and high probability density (HPD) for direct additive genetic ($\sigma_{a}^{2}$), residual ($\sigma_{e}^{2}$) variances and heritability (SD, standard deviation) for different categories of stayability in Nellore cattle.

Trait	$\sigma_{a}^{2}$	$\sigma_{e}^{2}$	h^2^	± SD	HPD
STAY48‐2	0.24	1.01	0.20	0.01	0.16 to 0.22
STAY48‐3	0.26	1.00	0.20	0.01	0.17 to 0.23
STAY54‐2	0.27	1.00	0.22	0.02	0.18 to 0.24
STAY54‐3	0.28	1.00	0.22	0.02	0.19 to 0.25
STAY72	0.19	1.00	0.16	0.01	0.14 to 0.18

*Note:* STAY48‐2: female's ability to remain in the herd up to 48 months of age. Calving to at least 2 times; STAY48‐3: female's ability to remain in the herd up to 48 months of age. Calving to at least 3 times. STAY54‐2: female's ability to remain in the herd up to 54 months of age. Calving to at least 2 times; STAY54‐3: female's ability to remain in the herd up to 54 months of age. Calving to at least 3 times and STAY72: female's ability to remain in the herd up to 72 months of age, calving to at least 3 times.

When STAY is evaluated based on the traditional definition, which considers survival until 72 months of age with at least three calvings per cow, this approach may not adequately reflect the probability of early calving at 24 and 26 months. In intensive production systems, heifers that calve for the first time in 24 months should reach up to five calvings by 72 months; otherwise, they may experience up to two reproductive failures during their productive lifespan, increasing the costs of maintaining nonproductive cows. Thus, the definition of STAY can influence the number of calves per cow, highlighting the need to develop an appropriate method for its measurement in early sexual precocious heifers (Bonamy et al. 2018). Additionally, stayability is a key fertility indicator, exhibiting a high genetic correlation with the female's ability to reconceive. This is evidenced by genetic correlations of 0.99 and 0.98 with the ability to produce consecutive calves at intervals shorter than 14 months for STAY52 and STAY72 respectively (Ramos et al. 2020).

Selection for STAY is challenging due to its low heritability, which typically ranges from 0.08 to 0.14 (Bonamy et al. 2018; Costa et al. 2020 and Kluska et al. 2018), and because it is a sex‐limited trait evaluated late in a female's life, usually at 72 months of age. These factors reduce the expected genetic gain when traditional STAY is included as a selection criterion in beef cattle breeding programs. Thus, selecting for this trait at earlier ages emerges as a strategy to increase the rate of genetic gain, based on the hypothesis that the main genetic factors influencing this trait act throughout the female's productive lifespan. Alternative STAY definitions that consider fewer reproductive failures may more accurately represent cow reproductive efficiency. Additionally, obtaining phenotypic records at earlier stages can accelerate genetic progress for both reproductive efficiency and longevity, enabling a more effective selection of animals with higher productive potential (Silva et al. 2024).

The posterior means, standard deviations and HPD intervals for the genetic correlations between different STAYs categories with growth, carcass, feed efficiency, fertility, body composition and sexual precocity‐related traits are presented in Tables 4, 5 and 6. The estimated genetic correlations between STAY54‐2 (Table 5), STAY54‐3 (Table 5), STAY72 (Table 6) and W240 were moderate to low and favourable, 0.28, 0.29 and 0.46 respectively. The correlations were higher with W240, and there may also have been an effect of selection or culling. On the other hand, the genetic correlations observed between STAY48‐2 (Table 4), STAY48‐3 (Table 4) and W240 were low, 0.17 and 0.18 respectively. Therefore, a small response can be expected from the selection of stay for weaning weight, highlighting that both traits should be used as selection criteria for greater genetic and productive gains in the weaning weight of the progeny and in the ability of the female to remain in the herd.

**TABLE 4:** Posterior mean, standard deviation (SD) and high probability density (HPD) for genetic correlation for STAY48–2 and 48–3, sexual precocity, fertility, growth, carcass, body composition and feed efficiency indicator traits in Nellore cattle.

Trait	Mean	± SD	HPD
Genetic correlation with STAY48‐2			
W240	0.17	0.04	0.08 to 0.25
W450	0.07	0.04	−0.01 to 0.15
DMI	0.08	0.09	−0.09 to 0.25
RFI	0.01	0.10	−0.17 to 0.23
RG	0.00	0.12	−0.21 to 0.24
REA	0.11	0.05	0.03 to 0.21
RFT	0.11	0.04	0.03 to 0.19
FRAME	−0.09	0.07	−0.22 to 0.04
PP30	0.93	0.01	0.91 to 0.95
STAY72	0.65	0.05	0.54 to 0.75
SC365	0.15	0.04	0.05 to 0.22
APM	−0.22	0.07	−0.35 to −0.07
Genetic correlation with STAY48‐3			
W240	0.18	0.04	0.09 to 0.26
W450	0.09	0.04	0.01 to 0.17
DMI	0.06	0.09	−0.09 to 0.24
RFI	0.00	0.10	−0.20 to 0.19
RG	−0.03	0.11	−0.25 to 0.18
REA	0.12	0.05	0.03 to 0.20
RFT	0.11	0.04	0.03 to 0.20
FRAME	−0.09	0.06	−0.21 to 0.03
PP30	0.90	0.01	0.87 to 0.92
STAY72	0.73	0.04	0.65 to 0.80
SC365	0.16	0.05	0.08 to 0.26
APM	−0.22	0.07	−0.36 to −0.08

**TABLE 5:** Posterior mean, standard deviation (SD) and high probability density (HPD) for genetic correlation for STAY54–2 and 54–3, sexual precocity, fertility, growth, carcass, body composition and feed efficiency indicator traits in Nellore cattle.

Trait	Mean	± SD	HPD
Genetic correlation with STAY54‐2			
W240	0.28	0.05	0.18 to 0.38
W450	0.14	0.04	0.06 to 0.22
DMI	0.13	0.08	−0.04 to 0.28
RFI	−0.03	0.09	−0.20 to 0.15
RG	0.09	0.13	−0.17 to 0.31
REA	0.18	0.04	0.09 to 0.25
RFT	0.12	0.04	0.04 to 0.20
FRAME	−0.06	0.07	−0.17 to 0.08
PP30	0.88	0.02	0.84 to 0.92
STAY72	0.68	0.05	0.57 to 0.77
SC365	0.18	0.04	0.09 to 0.26
APM	−0.18	0.08	−0.36 to −0.03
Genetic correlation with STAY54‐3			
W240	0.29	0.05	0.19 to 0.39
W450	0.14	0.04	0.06 to 0.23
DMI	0.11	0.08	−0.03 to 0.28
RFI	−0.03	0.10	−0.21 to 0.16
RG	0.04	0.10	−0.15 to 0.26
REA	0.12	0.05	0.02 to 0.22
RFT	0.12	0.04	0.03 to 0.20
FRAME	−0.07	0.06	−0.20 to 0.05
PP30	0.92	0.01	0.88 to 0.94
STAY72	0.69	0.04	0.60 to 0.77
SC365	0.19	0.04	0.09 to 0.27
APM	−0.20	0.07	−0.34 to −0.06

**TABLE 6:** Posterior mean, standard deviation (SD) and high probability density (HPD) for genetic correlation for STAY72–3, sexual precocity, fertility, growth, carcass, body composition and feed efficiency indicator traits in Nellore cattle.

Trait	Mean	± SD	HPD
Genetic correlation with STAY72			
W240	0.46	0.04	0.39 to 0.54
W450	0.34	0.04	0.27 to 0.42
DMI	−0.06	0.11	−0.28 to 0.15
RFI	−0.06	0.11	−0.26 to 0.14
RG	−0.07	0.14	−0.35 to 0.22
REA	0.31	0.05	0.21 to 0.40
RFT	0.18	0.05	0.09 to 0.28
FRAME	0.02	0.07	−0.09 to 0.17
PP30	0.28	0.06	0.16 to 0.40
SC365	0.32	0.04	0.23 to 0.41
APM	−0.30	0.09	−0.50 to −0.14

This cumulative weight is the sum of the calves that a dam has weaned throughout her productive life, for example, two or three calves. In addition, as previously discussed, this includes the female's ability to successfully impregnate, calve and raise calves until weaning, that is, kg of weaned calf, indirectly indicating the female's productive and reproductive efficiency. Females with higher weaning weights can also enter the reproductive season earlier (Baruselli et al. 2018). However, the low genetic correlation between having two or three calves by 48 months of age and weaning weight may be explained by the early culling of females that wean lighter calves—often after their second calving. This early removal from the herd can bias the genetic correlation estimate. In contrast, categories such as three calvings by 54 or 72 months of age more accurately reflect the female's lifetime productive potential. For W450, genetic correlations were low, with values ranging from 0.07 to 0.14 (Table 4), except the moderate correlation between STAY72 and W450 (0.34) (Table 6).

The feed efficiency indicator traits (RFI, DMI and RG) showed low and close to zero genetic correlations with the different selection criteria of STAY, ranging from −0.03 to 0.11, suggesting that selection for different STAY in early challenged heifers would not negatively affect feed efficiency traits or vice versa. Brunes et al. (2023) and Sainz et al. (2024) reported low genetic correlation estimates between STAY and RFI in Nellore cattle, with values of 0.14 and 0.12 respectively. Similar results between STAY and DMI (0.15) were reported by Sainz et al. (2024).

Using RFI as a selection criterion is proposed for extensive beef cattle breeding and calf production according to Brunes et al. (2023). The more efficient females tend to require lower maintenance requirements, which is a fundamental factor for females raised in more extensive systems since they make better use of the available natural resources, that is, better use of forage qualitatively and quantitatively, expressing fitness (greater adaptability). In this sense, these females are less susceptible to possible feed restrictions or feed shortages of protein and energy during the dry season, a common situation in pasture production systems of commercial and selective herds in central Brazil. Consequently, their reproductive efficiency and longevity are less affected by environmental conditions.

The different selection criteria of STAY (Tables 4, 5 and 6) showed low genetic correlations with REA and RFT, with values ranging from 0.11 to 0.18 and 0.11 to 0.12 respectively. Similar results were reported by Kluska et al. (2018) in Nellore cattle using the traditional STAY, with estimates ranging from 0.05 to 0.17 and 0.05 to 0.31 for REA and RFT, indicating that selection for improved carcass traits is unlikely to enhance cow longevity in the herd.

The estimated genetic correlation values between the different selection criteria of STAY (STAY48‐2, STAY48‐3, STAY54‐2 and STAY54‐3) and PPC30 (Tables 4, 5 and 6) were favourable and high: 0.93, 0.90, 0.88 and 0.92 respectively. On the other hand, as expected, the genetic similarity between STAY72 and PPC30 was moderate to low, which is in agreement with the findings of, Bonamy et al. (2018), which reported a moderate genetic correlation magnitude (0.42) between traditional STAY and PPC30 decreasing as a function of precocious calving age. Indirect selection for STAY through direct selection for PPC30 can result in more significant annual genetic gains. Genetic correlations between the different STAY approaches and traditional STAY had values ranging from 0.68 to 0.72. These values imply that selection for STAY measured earlier in the female's life can result in genetic gain for traditional STAY. In this sense, it can be stated that although traditional STAY can allow success even with two reproductive failures, it is not directly related to females who have reproductive failure in the initial phase of their reproductive cycle (Ramos et al. 2020).

Thus, the new STAY approaches reflect more directly the reproductive success of the dam, selected for heifers that have a greater probability of success in the initial phase of productive life, contributing to greater reproductive efficiency and lower cost per productive female. Furthermore, the probability of permanence can contribute positively to environmental sustainability, by reducing the unproductive phase of the life cycle, that is, reducing the period characterised by the development of heifers until the beginning of their productive life (Snelling et al. 2022). This reinforces that programs can use early permanence approaches to predict the reproductive efficiency of dams, better reflecting their reproductive performance and contributing to the economic and environmental sustainability of the production cycle given that the genetic correlation between the different selection criteria of STAY and PPC30 ranges from 0.88 to 0.93, whereas the traditional stayability shows a much lower value of only 0.28.

Reducing the calving age from 36 to 24 months led to a 20% increase in herd profitability and a 33% decrease in replacement rate, as demonstrated by Roy (1976) in dairy cattle. These results highlight the importance of selecting earlier‐challenged females to reduce replacement costs and enhance the system's productive efficiency. However, to optimise this strategy in beef cattle, it is essential to assess female longevity based on their reproductive performance throughout life rather than relying exclusively on traditional STAY, evaluated at 72 months of age.

The new approach of the study and inclusion of different selection criteria of STAY is because Zebu females are challenged at younger ages in the reproductive season due to the increasing selection for sexual precocity in the last 14 years, especially in females of the Nellore breed. On farms where early or precocious females are challenged, the first calving occurs between 24 and 30 months of age, and these females are expected to produce five calves by 72 months of age. If they fail, this female has failed reproductively twice, consequently increasing the additional costs of unproductive categories (Bonamy et al. 2018). However, considering the physiological stage of females at this age, the traditional STAY may not be efficient for this category.

For a female to be truly efficient, it is not enough to conceive early, but also reconceive in the following season without long postpartum anestrus (Ramos et al. 2020). However, multiple factors such as environment, feed management and nonadditive genetic effects can hinder reconception in early‐calving heifers (Bonamy et al. 2018). The added physiological demands of lactation and growth also contribute to lower reconception rates in this group. As a result, the number of calves per cow can vary depending on how STAY is defined, and the traditional definition may penalise these early‐calving females (Bonamy et al. 2018). Based on the genetic correlations in this study, alternative STAY definitions may better capture fertility in females with shorter reproductive cycles and better reconception ability. The genetic correlation between FRAME and the different categories of STAY was low, ranging from −0.09 to −0.06, that is, selection for STAY would not affect the selection response to FRAME. Negreiros et al. (2022) reported similar results in Nellore cattle. With a genetic correlation of 0.06 for traditional STAY, and this low correlation may reflect the distinct management practices of the analysed herds. In this context, considering the different regions and production systems, it is important to emphasise that the body composition or FRAME of the animals must be aligned with the selection criteria and objectives, respecting the breeding environment.

This study obtained low and favourable genetic correlations between different categories of STAY and SC365 (ranging from 0.15 to 0.19) and STAY and APM (ranging from −0.22 to −0.18) (Tables 4, 5 and 6). The genetic correlation with SC365 was similar to that found by Costa et al. (2020) (0.12). Silva Neto et al. (2020) reported a moderate genetic correlation between STAY and APM (−0.35), differing from the results of the present study. According to the authors, STAY is an economically important trait and, when included in breeding programs, can enable the selection of sires whose daughters have a higher probability of remaining productive in the herd for a more extended period.

## 4 Conclusion

The results of this study demonstrate that the different stayability categories (STAY48‐2, STAY48‐3, STAY54‐2 and STAY54‐3) present genetic variability and can contribute to genetic progress, supporting their use as selection criteria for longevity in herds where the heifers are first exposed to first mating at ages below 21 months of age. High and favourable genetic correlations with sexual precocity traits, such as PPC30, reinforce their potential in genetic improvement programs to accelerate gains in reproductive performance and enhance the economic sustainability of cow–calf operations with early‐challenge systems for young females.

The choice of stayability category should align with the selection objective of the production system. Stayability, measured as giving birth to two calves in 48 months, offers the advantage of early measurement, enabling faster genetic progress due to a shorter generation interval. However, it prioritises reconception ability over long‐term productive efficiency. In contrast, stayability, defined as giving birth to three calves by 54 months, provides a more comprehensive assessment of real productivity and longevity, despite being a more demanding criterion. Genetically, differences between these stayability categories are small, meaning the decision on which to use should be guided primarily by production goals and zootechnical considerations.

## Ethics Statement

This study was exempt from evaluation by the Animal Ethics Committee (CEUA), as established by Law No. 11,794 of 08/10/2008 and Normative Resolution No. 51 of 05/19/2021 from the National Council for Animal Experimentation Control (CONCEA), because the data were obtained from an existing database.

## Conflicts of Interest

The authors declare no conflicts of interest.

## Data Availability

The data that support the findings of this study are available on request from the corresponding author. The data are not publicly available due to privacy or ethical restrictions.
